# Use of Biomass Bottom Ash as an Alternative Solution to Natural Aggregates in Concrete Applications: A Review

**DOI:** 10.3390/ma17184504

**Published:** 2024-09-13

**Authors:** Florian Schlupp, Jonathan Page, Chafika Djelal, Laurent Libessart

**Affiliations:** Univ. Artois, IMT Nord Europe, Junia, Univ. Lille, ULR 4515, Laboratoire de Génie Civil et géo-Environnement (LGCgE), F-62400 Béthune, France; jonathan.page@univ-artois.fr (J.P.);

**Keywords:** solid waste, sand replacement, sustainable, waste management, recovery and recycling, circular economy

## Abstract

Biomass bottom ash (BBA) is a by-product of the energy industry and is produced from biomass-fired thermal power plants. They represent the coarsest fraction of the recovered ash and are mostly landfilled. Several researchers have investigated the feasibility of the use of BBA as a replacement for natural aggregates in cementitious material. The utilisation of BBA in the manufacturing of concrete provides an economic and ecological way to upcycle it. At the same time, its use conserves natural resources and promotes sustainability. This review article first presents the chemical, mineralogical and physical properties of BBA, to highlight the possible effects on cementitious materials and the interest in valorising them as a building material. Secondly, the focus is on the utilisation of BBA incorporated in place of natural aggregates used in the manufacturing of concrete. This review investigates the multi-physical properties of concrete manufactured with the partial incorporation of BBA. This substitution leads to decreased workability, which can be limited by the use of admixtures. In the hardened state, a reduction in the mechanical properties is shown with BBA replacement. However, many experimental works show that BBA can be used in appropriate proportions to maintain the specified properties of the concrete.

## 1. Introduction

The development of renewable energy production at a European level is set to increase in the future. The Renewable Energy Directive (RED I) [1] had already set a production target of 20% renewable energy by 2020; the revision of the directive in 2018 (RED II) increased this target to 32% by 2030 [2]. These directives will shift the European energy mix towards more sustainable energy solutions: photovoltaic, wind, biomass and hydro. The development of the biomass sector for electricity and heat production will lead to an increase in the production of ash related to these technologies. The amount of biomass ash related to energy production worldwide is estimated at about 170 million tonnes per year and will increase in the future [3]. This represents a significant amount of waste to be recovered or disposed of by operators. Biomass ash (BA) is usually sent to landfill; however, this solution has high costs, which can reach up to EUR 500 per ton in Europe [4]. Some BA can be used as agricultural or forestry fertiliser, but the level of heavy metals and the pH can limit this use [4,5]. An interesting avenue of utilisation is the use of biomass bottom ash (BBA), which is generally coarser than biomass fly ash (BFA), as a granular supplement or granular substitute in the construction industry.

In addition to the production of biomass ash, the global consumption of sand related to the production of concrete, glass and electronics is estimated to be between 32 and 50 billion tonnes consumed per year [6,7]. Projections of the global evolution of sand use and availability show that, by 2020, the demand will exceed production [8]. Recent studies seem to confirm that the demand for construction sand is likely to increase by 45% over the next few decades [9,10]. In addition to the need for raw materials, sand extraction also contributes to changes in biodiversity, landscapes, water flows in the soil and the local climate. There are also socio-economic, cultural and political impacts. These effects are all the more marked when there is no regulation in place [11]. It is therefore important to find a sustainable alternative to these raw materials.

Increasingly, the use of coal for energy production is being reduced in favour of renewable energy in the European Union [12]. Coal ash is already valorised (EN 197-1 [13]; EN 450-1 [14]) and studied in the literature for cement and granular substitution in cementitious materials [15,16]. As with coal ash, biomass ash has interesting potential for the production of cementitious composites. However, the physical and chemical properties of biomass ash differ from those of coal ash [3].

Literature reviews on the incorporation of biomass ash into cementitious materials generally focus on the replacement of cement with fly ash [17,18]. The particularity of the present review is that the focus here is on the potential utilisation of biomass ash—specifically bottom ash—as a granular substitute in cementitious materials. Bottom ash is coarser and generally less reactive than fly ash, so a possible recycling option could be to incorporate it as an aggregate in cementitious materials. Consequently, studies on the properties and incorporation of BBA into mortar and concrete have been used in this review. It is based on 47 studies published in the literature on biomass ash, including 41 presenting the properties of the ash and 18 studies on aggregate substitutions. Studies on the incorporation of coal ash and biomass fly ash are used to corroborate some of the findings. The development of the review is separated into two parts: the first part presents biomass bottom ash (BBA) used as a construction material (origins, physico-chemical properties and ash processing) and the second part deals with the valorisation of BBA as an aggregate in cementitious materials (fresh and hardened properties, durability).

## 2. Origin and Properties of Biomass Ash (BA)

Directive 2009/28/EC of the European Parliament and of the Council gives a general definition of biomass: “the biodegradable fraction of products, waste and residues from biological origin from agriculture (including vegetal and animal substances), forestry and related industries including fisheries and aquaculture, as well as the biodegradable fraction of industrial and municipal waste” [1]. In order to successfully develop the properties of biomass ash (BA), it is important to understand the phenomena involved in and influencing its production. These range from the combustion process to the main technological principles.

### 2.1. Origin and Production of Biomass Ash

**Figure 1:**
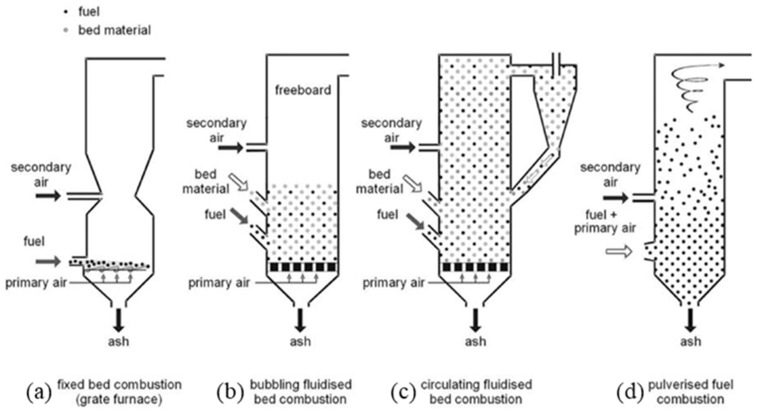
Combustion technology (from [19]).

The properties of biomass ash (BA) mainly differ according to the combustion technology used and the origin of the biomass. Different technologies are used for biomass combustion, and, among them, three are mainly used on an industrial scale: fixed bed combustion (a), bubbling fluidised bed combustion (b) and circulating (c) and pulverised fuel combustion (d) (Figure 1) [19,20]. The main difference between these technologies is the combustion zone. In the case of fixed bed technology, combustion takes place on the bed material. In fluidised bed technology, a bed material (silica sand or dolomite) is introduced, kept in suspension by a gas flow, and combustion takes place on it. The difference between dense fluidised bed and circulating fluidised bed technology is the speed of gas introduction, which allows the suspension or circulation of the biomass/bed material mixture. The unique feature of pulverised biomass technology is that combustion takes place when the biomass is in suspension. For each technology, the addition of a primary and secondary airstream completes the combustion.

**Figure 2:**
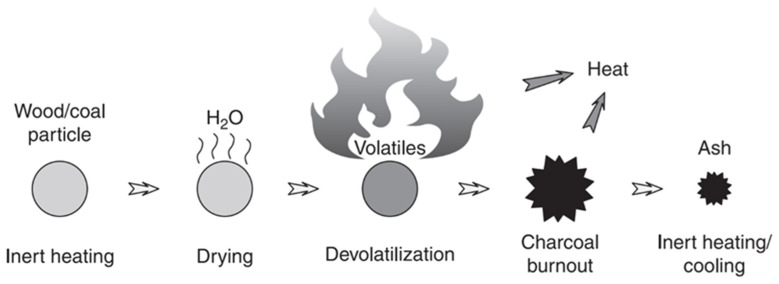
Combustion process of a particle (from [20]).

During the combustion process, different phases can be observed in the transformation of the biomass particle into ash. These different phases are drying, pyrolysis, gasification and combustion (Figure 2) [19,20]. The production of ash as a result of these reactions is influenced by the combustion technology and, in particular, by the efficiency of the combustion technology to achieve the most complete combustion possible. In the case of fluidised bed technologies, the presence of a bed material (usually sand) causes the more complex adhesion and fusion of the biomass with the bed material [21,22,23,24,25].

**Figure 3:**
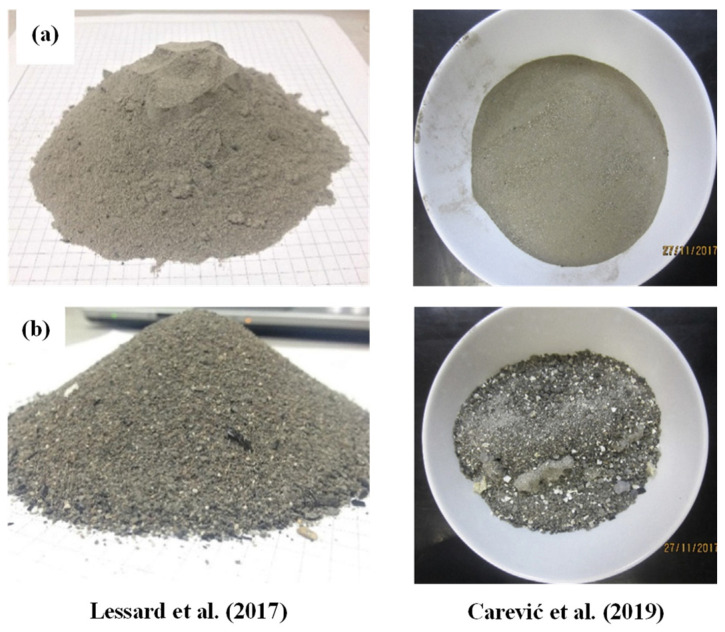
Bulk appearance of fly ash (line **a**) and bottom ash (line **b**) (from [4,26]).

Following combustion, there are two main categories of ash:

- Fly ash (Figure 3, line a)—this is recovered during flue gas treatment operations.
- Bottom ash (Figure 3, line b)—this is the coarsest fraction of the recovered ash and is derived from the non-volatile ash.

**Figure 4:**
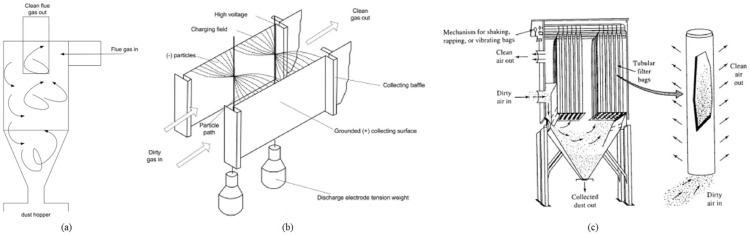
Operation of the main types of filters: (**a**) cyclonic filter, (**b**) electrostatic filter, (**c**) bag filter (from [19]).

The properties of fly ash are influenced by the filtration method used (Figure 4). Indeed, the performance of the filters depends on the size of the ash particles to be recovered. A cyclone filter (a) performs better with coarse fly ash compared to electrostatic filters (b) or bag filters (c) [19,27]. The literature groups together coarse ash, recovered from the underside of the combustion chamber, regardless of the combustion technology used. Thus, bottom ash refers to sand that has undergone combustion via fluidised bed technology [25] and the coarse fraction of ash or “agglomerates” recovered from other combustion technologies (fixed bed or pulverised fuel combustion).

The local availability of biomass resources and the industrial profile of the region determine the type of biomass used in the combustion process. For example, biomass from the palm oil industry and rice cultivation is widely used in Asia [28,29,30]. In Brazil, biomass from sugarcane fields is predominant [31], while, in Canada, biomass from the pulp industry is used [26,32]. In Europe, wood biomass is more commonly used [4,33,34,35]. The origin of the biomass is a key parameter that can influence the ash properties [4,36,37,38]. The properties of the biomass influence the properties of the ash [39]; therefore, it is necessary to study the properties of the biomass prior to its use.

#### 2.1.1. Chemical Composition

The study of Vassilev et al. (2010), which analysed more than 86 biomass ashes, shows that the proportions of the most abundant oxides change depending on the origin of the biomass [39]:

- CaO (43.03%) > SiO_2_ (22.22%) > K_2_O (10.75%) > MgO (6.07%) for wood-based biomass;
- SiO_2_ (47.02%) > K_2_O (24.55%) > CaO (12.61%) > MgO (5.44%) for biomass from herbaceous straw and agriculture;
- K_2_O (28.25%) > SiO_2_ (24.47%) > CaO (16.58%) > P_2_O_5_ (7.27%) for biomass from grass and agricultural residues;
- SiO_2_ (35.73%) > CaO (18.30%) > Al_2_O_3_ (15.41%) > P_2_O_5_ (3.64%) for contaminated biomass.

Similar chemical compositions have been reported by authors working on wood biomass [4]. The origin of the biomass alone does not allow us to generalise about the characteristics of biomass ash. In fact, there are significant variations in the chemical composition of the different biomass families, linked in particular to the different combustion technologies used in the industry [4,19,39].

**Figure 5:**
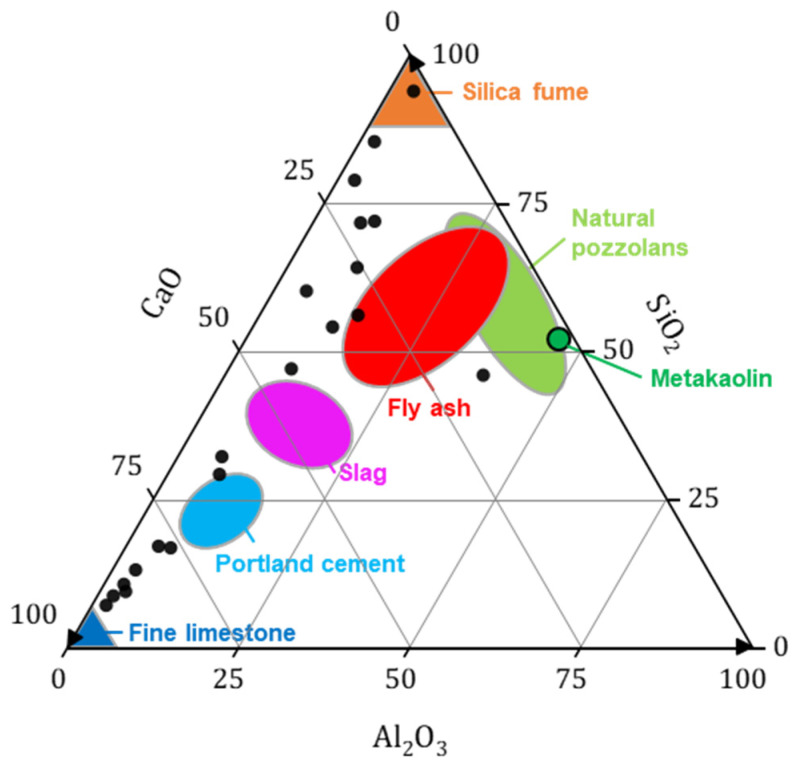
Ternary diagram of CaO-Al_2_O_3_-SiO_2_ of BBA (black dots) and supplementary cementitious materials according to Lothenbach et al. 2011 [48].

In order to compare biomass bottom ash (20 samples from the literature) with other supplementary cementing materials, a representation in the form of a CaO-Al_2_O_3_-SiO_2_ ternary diagram is proposed in Figure 5 [4,25,26,27,40,41,42,43,44,45]. Most of the BBA is located between Portland cement and a limestone filler. The strong dispersion of the ash is observed in the literature between the CaO and SiO_2_ content, which is mainly due to the combustion technology used. Indeed, ash with significant SiO_2_ content is generally derived from fluidised bed plants, as they use boiler sand in their combustion process [4,38,42,46,47].

**Figure 6:**
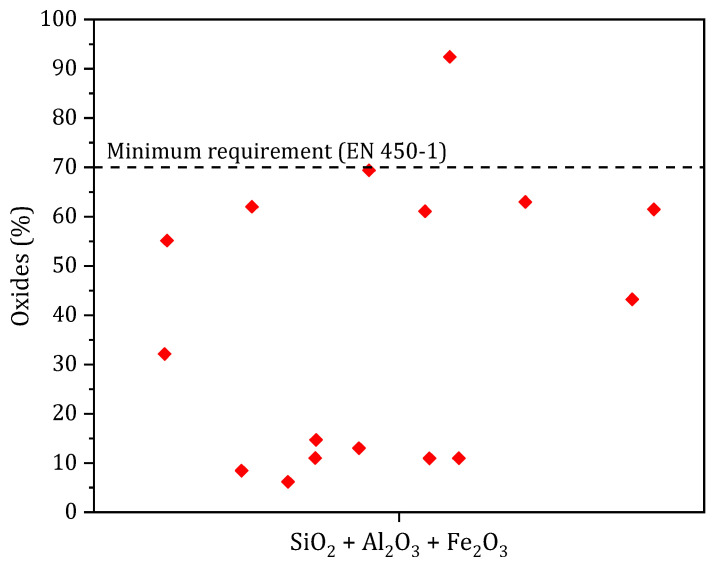
Sum of pozzolanic oxides (SiO_2_ + Al_2_O_3_ + Fe_2_O_3_) according to BBA from literature and minimum requirement (EN 450-1).

The presence of silica (SiO_2_), alumina (Al_2_O_3_) and ferrous oxide (Fe_2_O_3_) in biomass ash makes it a potential candidate for pozzolanic or hydraulic components in the cement matrix. The pozzolanic activity, expressed as the sum of pozzolanic oxides (SiO_2_ + Al_2_O_3_ + Fe_2_O_3_) according to EN 450-1 [14], must be higher than 70%. Figure 6 shows the sum of pozzolanic oxides from studies on BBA; most studies show that this threshold is not reached for biomass ash [4,25,26,27,40,41,43,44,45]. Only one sample of BBA has a value higher than or equal to the minimum required, six samples of BBA have values between 50 and 70% and 11 samples have values of less than 50%.

Due to its chemical composition, BBA generally has inert behaviour when it replaces part of the cement.

Sodium oxide (Na_2_O) and potassium oxide (K_2_O), expressed as Na_2_O equivalents (or Na_2_O_eq_), are present with generally higher content in fly ash than in sub-furnace ash. The observed difference in the value of sub-furnace ash is attributed to the combustion system [4]. More generally, ash from wood combustion has higher alkali content than coal ash [49]. The literature shows that the proportions of Na_2_O_eq_ are higher than the limit imposed by the EN 450-1 standard, which sets the presence of these oxides at less than 5% by mass to limit the negative effects on the durability of the materials [4,50].

The loss on ignition of BBA, ranging from 0.98 to 41.49% (Table 1), is higher than the loss on ignition of coal bottom ash, which ranges from 0.10 to 8.10% [15]. It represents the unburnt and organic elements present in the ash. The loss on ignition is an important parameter in the formulation of mortar and concrete since these particles absorb significant amounts of water and admixtures, resulting in reduced workability, lower mechanical strength and impacts on the durability of the cementitious materials produced [45,51]. Unsuitable burning processes [52,53] and insufficient burning temperatures [30] are factors that increase the loss on ignition content in the ash.

#### 2.1.2. Heavy Metals

Furthermore, the presence of heavy metals (Cd, Cr, Cu, Pb, Ni, Zn, Hg) in biomass ash is a factor limiting its recovery in the agricultural industry [4]. High levels of heavy metals can contaminate soil and be toxic to living organisms. The European classification classifies wastes according to their hazardousness in terms of the number of heavy metals present in three classes: inert, non-hazardous and hazardous [1]. Several studies show that most metallic elements do not exceed the thresholds for classification as inert waste and, in some cases, as non-hazardous waste [4,54,55,56]. The method of ash recovery will play a role in the proportion of heavy metals in the ash. Most of the heavy metals in fly ash come from cyclone filters. The residence time and high temperatures in the boiler are responsible for the high number of heavy metals in fly ash [27]. The presence of elements like Cu, As, Cr and Pb is mainly due to agricultural pesticides [54,57]. Leaching tests carried out on mortar samples have shown that when biomass ash is incorporated, the concentration of heavy metals decreases [56,58].

#### 2.1.3. Mineralogical Composition

The mineralogical characterisation of biomass ash shows a significant disparity in the literature. Indeed, some phases are more common, such as quartz, calcite, sylvite, arcanite, anhydrite, char, glass, lime, periclase and hematite. Meanwhile, others include portlandite, cristobalite, hydroxylapatite, larnite, albite, Ca phosphates, fairchildite, K carbonate, K feldspars, halite and K–Ca silicates [4,38,39,42]. They are mainly influenced by the type of biomass used (wood, bark, rice husks, sugarcane bagasse, etc.), but also by the combustion method (grate furnace, fluidised bed, pulverised biomass) and by the ash recovery process. The study of Carević et al. (2019) focused on the mineralogy of biomass ash from different combustion technologies [4]. It highlighted that BBA showed predominantly crystalline peaks and the absence of an amorphous phase. A mineralogical analysis showed that almost all ash analysed had peaks relating to calcium oxides (CaO) and calcium carbonates (CaCO_3_).

The presence of quartz seems to represent the majority in the ash from fluidised bed plants as silica sand is generally used in the combustion process [4,19,38], while the presence of other elements seems to be more dependent on the fuel used. The reactivity of the ash in the case of cementitious valorisation is limited by the lack of amorphous qualities in the ash. However, the presence of crystallised elements can be interesting in terms of granular valorisation.

#### 2.1.4. Morphology

**Figure 7:**
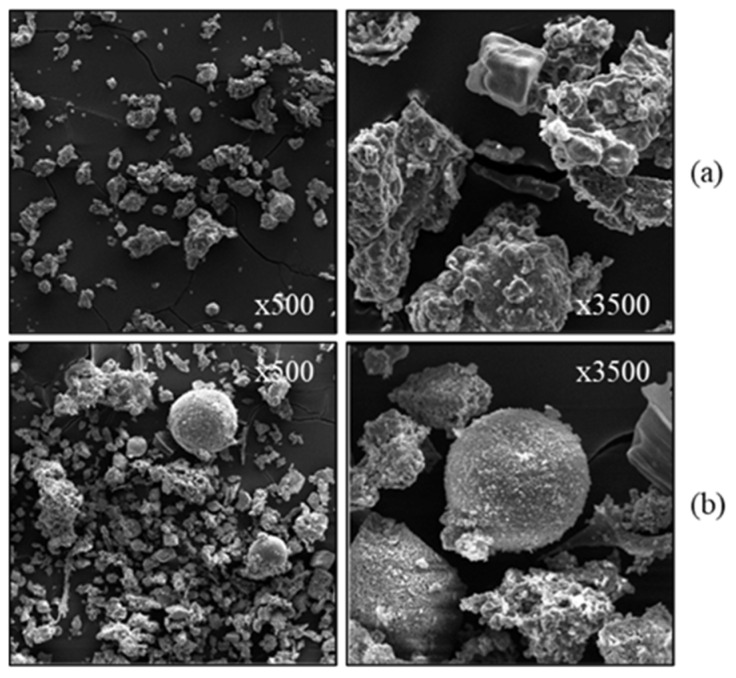
SEM observations of BBA from different sources: (**a**) grid technology, (**b**) pulverised fuel technology (from [4]).

Generally irregular in shape and size with inhomogeneous surfaces, BBA can also be porous and angular [4,26,27,38,43]. The morphologies of two types of BBA are shown in Figure 7. Figure 7a shows two observations of BBA particles from grate combustion technology. The particles have a textured and angular appearance. Figure 7b shows two observations of BBA particles from pulverised biomass technology. Different particle shapes are observed, sometimes rounded and textured [4]. Unburnt particles are also present in BBA; they generally have an elongated shape and a porous structure and are very friable [26]. Comparing the structures of biomass bottom ash with those from coal combustion, similarities can be highlighted, such as their angular, heterogeneous and porous shapes [15,59,60]. The shape irregularities and porosity of BBA particles will cause changes in their water uptake. This could influence the water demand and thus negatively affect the workability of cementitious materials [26].

#### 2.1.5. Density and Water Absorption

The density properties and water absorption of BBA show large fluctuations between the different studies presented in Table 1. The density varies between 1.73 and 2.69 g/cm^3^ and water absorption between 5.2 and 39.3%. These variations are significant for the different combustion methods and biomass types used. The presence of loss on ignition (LOI) related to the efficiency of the combustion method varies between 0.98 and 41.49% in BBA. These lower-density particles tend to contribute to the decrease in density and could increase the absorption of water. The influence of the boiler efficiency also plays a role in the density variability. Incomplete combustion tends to produce more unburnt material, which in turn decreases the density [45,53].

**Table 1:** Density, water absorption and LOI of BBA.

Type of Biomass	Combustion Technology	Density (g/cm^3^)	Water Absorption (%)	LOI (%)	Source
Wood	Fluidised bed	2.28	2.41	1.84	[25]
Wood	Fluidised bed	2.65 to 2.67	n.a.	1.27 to 2.08	[42]
Wood	Multiple	1.75 to 2.69	n.a.	1.00 to 19.60	[4]
Agricultural	n.a.	2.02	19.90	3.06	[61]
Agricultural	n.a.	1.89 to 2.01	19.5 to 19.8	3.41 to 3.92	[44]
Agricultural	Grid	2.21 to 2.49	19.60 to 39.30	11.00 to 16.86	[52]
Agricultural	Grid	2.35	n.a.	41.49	[43]
Agricultural /wood	n.a.	2.34 to 2.41	n.a.	n.a.	[41]
Agricultural /wood	Grid	1.97	26.60	4.12	[55]
Agricultural /wood	Grid	1.82	31.90	4.85	[62]
Agricultural /wood	Grid	1.86 to 2.12	18.90 to 21.80	0.98 to 4.34	[45]
Agricultural /wood	Grid	1.73	19.83	n.a.	[40]
Agricultural /wood	Grid	2.34 to 2.38	9.80 to 10.10	n.a.	[54]
Pulp and paper industry	Fluidised bed	2.21	5.20	1.90	[26]

n.a.: not available.

In the case of fluidised bed technologies, the presence of cracks and surface porosity in the particles may explain the increased water absorption. The presence of sand in the recovered ash could explain the lower water absorption compared with other combustion technologies [25]. Compared to the density and water absorption of conventional construction sand, which are 2.6 g/cm^3^ and 1–4%, respectively, BBA has significant water absorption. This may affect the workability and hydration reactions by decreasing the water/cement ratio of cementitious composite formulations.

#### 2.1.6. Granular Distribution

**Figure 8:**
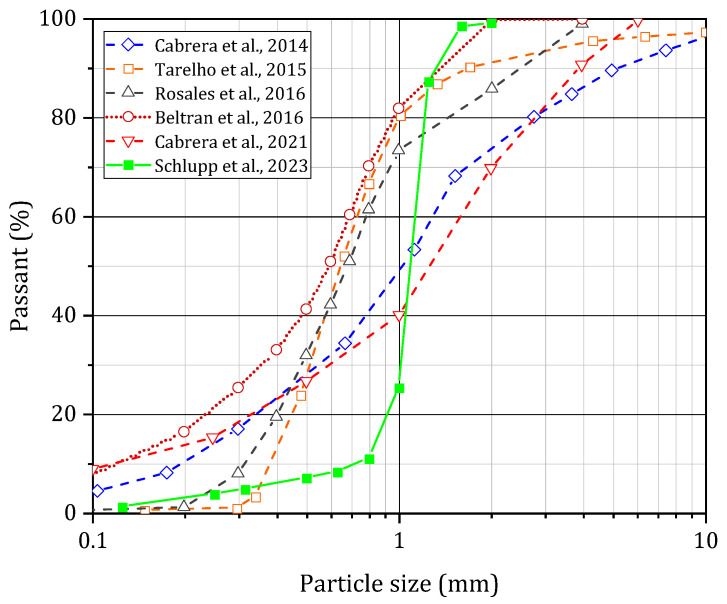
Different grain sizes of bottom ash according to the literature [38,40,47,55,63,64].

The particle size of BBA also depends on the combustion technology used; thus, fluidised bed ash will have a particle size close to that of the bed material used in the power plant [4,26,41,46]. Typically, their particle size is between 0.063 and 4 mm with high fine element content [4,62]. The presence of undesirable elements in the biomass, such as metallic elements, soil and gravel, causes the particle size to vary beyond 4 mm. The particle size curves of several BBA samples are presented in Figure 8 [38,40,47,55,63,64]. The median ash diameter varies between 0.59 and 1.27 mm. The modulus of fineness calculated according to EN 12620 [65] is between 2.46 and 3.61 for the different ashes studied. The majority of the ashes are classified as coarse-grained sands. The grain distribution of the BBA seems to be influenced by the presence of undesirable elements in the biomass used in the combustion process. For ash from fluidised bed technology, the grain size distribution is also influenced by the bed material used for combustion [47].

### 2.2. Improvement of Ash Properties

The presence of undesirable elements such as light particles, organic matter or unburnt elements in biomass ash can restrict the possibilities of its recovery in cementitious materials. The use of pre-treatment on the ash, such as grinding, combustion, the removal of light particles, sieving and washing, can increase the recoverable part of the ash. Various studies have investigated the benefits of these treatments for biomass ash [42,45,66].

The research of Modolo et al. (2013) focused on the washing of bottom ash in order to reduce the number of soluble chloride salts in it [42]. This can lead to potential corrosion where steel-reinforced constructions are involved. The industrial washing process, also used to wash materials for the construction sector, consists of passing the material under a shower with a liquid/solid flow rate of 2. The results of the study show a decrease in the number of chlorides of more than 66% compared to the amount initially present in the ash.

Rosales et al. (2017) studied different ash treatment processes for use as a cement substitute: grinding, combustion, light particle removal and the combination of these treatments [45]. The different treatments tended to change the particle size of the ash to approach that of cement (1–100 μm). Burning the ash at 800 °C reduced the amount of organic matter by 77% and was the most efficient compared to the other methods; similar observations were obtained by other authors for coal ash [53]. The water uptake of the ash decreases as a result of the treatment, which is related to the decrease in the number of light particles, which is reduced with the treatment. Agrela et al. (2017) report similar findings on BBA properties with a light particle removal treatment; the results on the mechanical properties and durability of concrete are less significant [66].

The combination of different treatments makes it possible to obtain a clear improvement in the properties of mortars incorporating ash as a replacement for cement. They can achieve 28-day strength gains ranging from 3 to 27 MPa depending on the treatment or combination of treatments compared to untreated ash [45]. However, the studies presenting the use of BBA as sand show marginal or low improvements when the treatment is applied [42,66]. In addition, it must be taken into account that the use of a pre-treatment on the ash impacts the material’s environmental performance. This can reduce the environmental potential of ash compared with other materials, although this impact is less significant than the extraction of natural resources and the production of traditional cement [67]. Ash treatment should be used in moderation to avoid additional financial and environmental costs, particularly when the treatment has no significant effect on the properties of cementitious materials.

### 2.3. Interest in and Possibility of Valorisation

In summary, BBA is a material generally composed of silica, calcium oxide and potassium oxide. Its mineralogy consists mainly of crystallised elements and no amorphous phase. The grain size distribution of BBA is frequently 0/4 mm and it can be characterised as coarse-grained sand. The density of BBA varies between 1.73 and 2.69 g/cm^3^ and its water absorption is commonly above 10%.

The amorphous nature, the difference in particle size compared to cement and the insufficient presence of potentially reactive elements in BBA limit its use as a cement substitute in cementitious composites. In addition, the high water absorption and the presence of unburnt particles also limit its use.

However, the grain size distribution of BBA, being between 0 and 4 mm, makes it favourable to use ash as a granular component of a mix, despite the presence of fines. The grain size of most BBA types remains close to that of construction sand. The use of ash as sand can increase the recoverable portion in concrete.

Furthermore, the environmental impact of using biomass ash as a building material is better than that of landfilling it. It reduces the extraction and processing of natural raw materials [67].

## 3. Influence of Granular Substitution of Biomass Ash on Cementitious Materials

Several authors have investigated the incorporation of biomass ash as a granular material in the production of mortars and concretes. Their studies show that the use of biomass ash results in lower workability and mechanical strength than the control formulation. This section proposes to link the properties of the ash to changes in the properties of concrete.

### 3.1. Workability

Generally, the incorporation of biomass ash in cementitious materials as sand leads to a decrease in workability. Indeed, the high water absorption of these ashes, linked to their morphology and high loss on ignition, is responsible for the observed decrease in workability. In addition, the finer modulus of ash compared to the sand used can increase the amount of water needed to lubricate the grains, which also impacts the workability [26,61,63]. Studies show that it is possible to maintain constant workability in the fresh state with the use of admixtures, without problems of interaction between the ash and admixture [26,44,55,61,63,66,68]. The majority of studies use W/C ratios between 0.50 and 0.60, with the amount of admixture varying from 2.1 to 8.4 kg/m^3^, usually water-reducing superplasticisers, in order to maintain a similar consistency to the reference formulations. It is also pointed out that the substitution of sand by BBA by mass with density differences will create a greater difference in volume, which accentuates the effects of the loss of workability [26].

### 3.2. Fresh Density

**Figure 9:**
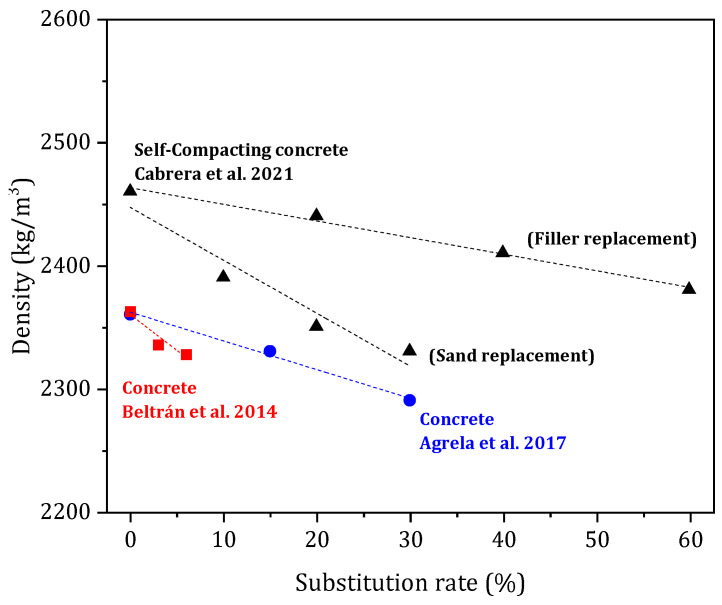
Concrete formulation density compared to the substitution rate [40,61,66].

The density of mortar and concrete formulations decreases with the substitution rate of biomass ash. Figure 9 shows the density evolution of concrete from different studies for sand substitution rates of 0 to 30% and filler substitution of 0 to 60%. Compared to the reference material, biomass ash has a lower density and higher water absorption, resulting in a decrease in the density of the cementitious composites [40,54,55,61,63,66]. For mortar formulations, the authors observed an 8–10% decrease in density. For substitutions of less than 30% in concrete formulations, the observed decreases are relatively small, 1 to 5%, compared to the reference. The biomass ash in these formulations represents 1 to 16% of the aggregates used, which may explain the small decrease in density observed by the authors. In the case of filler substitution, a smaller decrease in concrete density is visible due to the lower amount of ash substituted in the concrete mix design compared to sand substitutions.

### 3.3. Mechanical Resistance

#### 3.3.1. Compressive Strength

**Figure 10:**
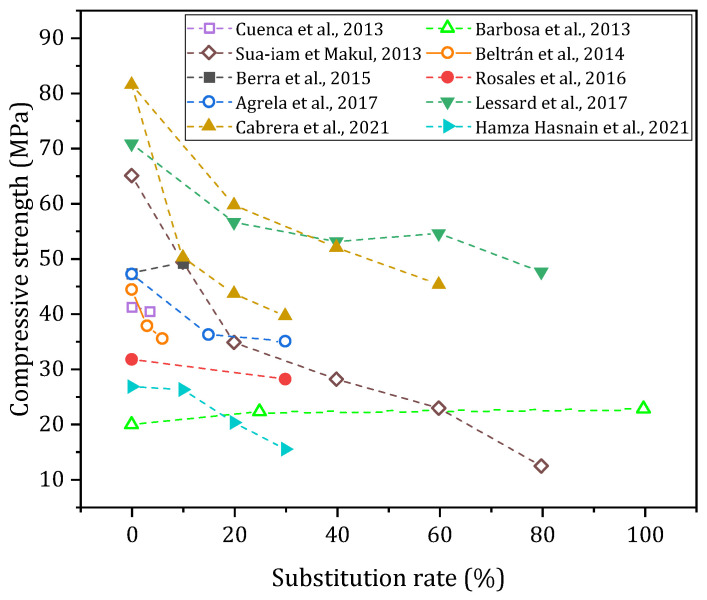
Compressive strength (28 days) according to the literature [26,35,40,55,61,66,68,70,71,72].

The effects of using biomass ash as an aggregate and filler in cementitious materials have been studied by different authors at the mortar scale [25,42,44,47,63,69] and on different types of concrete [26,31,35,40,54,55,61,63,66,68,70,71,72]. The results of the different studies are presented in Figure 10 (concrete).

The research by Modolo et al. (2013) studied the effects of incorporating BBA from a fluidised bed plant into bonding mortars. They showed that it is possible to replace up to 30% of the sand in the mix while maintaining mechanical strength equivalent to that of the reference. The composition of the ash, as well as the granular distribution close to that of the reference sand, allowed these results to be achieved [42,69]. The studies published by Schlupp et al. (2023) on BBA from fluidised bed power plants show that this ash is mainly composed of sand from the combustion technology. The study highlights the fact that it is possible to use 100% of the BBA as a sand to mortar with a gain in compressive strength compared to sand of a similar particle size [25,47]. Ash from fluidised beds is still not widely studied in the literature, even though it could have a positive or neutral impact on the properties of cementitious materials. In the case of other studies, the substitution of 10–20% of sand with BBA in the studies of Beltrán et al. (2016) and Rosales et al. (2023) led to decreases ranging from 7 to 57% depending on the formulation [44,63].

The use of biomass ash as sand on the concrete scale generally leads to a decrease in mechanical strength. Studies published by Beltrán et al. (2014) in the manufacture of plastic consistency concrete show a decrease in compressive strength for substitution rates of 3–6%. This is especially due to the significant water absorption of ash related to the presence of unburnt material [61]. The thesis work of Lessard (2017), with bottom ash as a substitute for sand in dry concrete, presents similar conclusions to the previous studies. The incorporation of 20–80% ash tends to decrease the mechanical strength by 20–30% compared to the reference [26]. Recent work by Cabrera et al. (2021) on the substitution of filler (20–60%) and sand (10–30%) in self-compacting concretes shows similar results, with a decrease in strength that varies between 25 and 50%. In contrast, studies by Berra et al. (2015) and Rosales et al. (2016) show that neutral results can be obtained when incorporating 10–30% of biomass ash in concrete. The proximity between the grain sizes of the biomass ash and the sand used, as well as the decrease in effective water due to the absorption of biomass ash, may be responsible for these effects [55,68]. The chemical reaction hypothesis studied by Berra et al. (2015) shows that a weak hydraulic reaction takes place when ash is incorporated into the cementitious matrix, without having a significant effect on the mechanical strength [68]. Similar results were obtained in the manufacture of self-compacting concrete by Cuenca et al. (2013) [35].

#### 3.3.2. Elastic Modulus

The effects of BBA substitution on the elastic modulus have rarely been studied in the various research works; only Cabrera et al. (2021) propose this test on concrete [40]. Tests carried out according to EN 12390-13 [73] show a 25 to 49% decrease in the elastic modulus with the incorporation of 20 to 60% BBA as a filler or 10 to 30% as sand. These results can be correlated with the previously mentioned decreases in compressive strength related to the incorporation of BBA in concrete.

#### 3.3.3. Flexural and Tensile Strength

**Figure 11:**
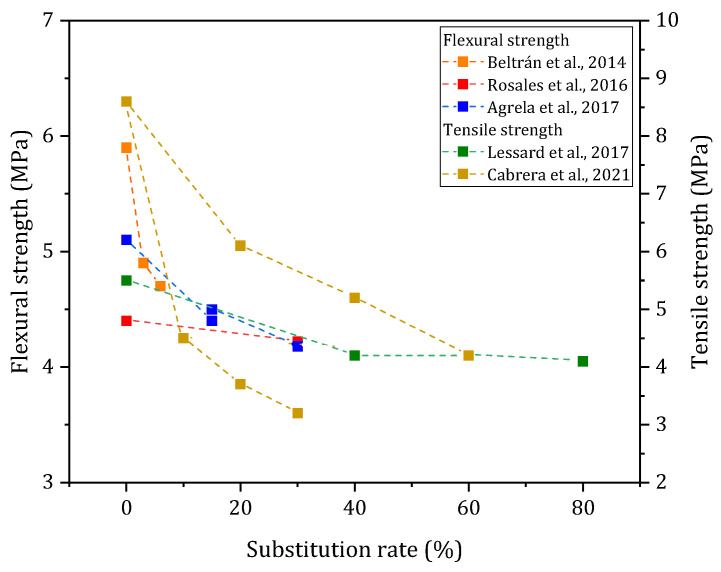
Flexural and tensile strength (28 days) according to the literature [26,40,55,61,66].

The flexural [55,61,66] and splitting tensile strength [26,40] are investigated in several works on the concrete scale; the results are presented in Figure 11. In the different studies, the incorporation of BBA as a sand or filler decreases the flexural and tensile strength and is dependent on parameters similar to those described for the compressive strength.

### 3.4. Durability

A concrete structure must resist various stresses and strains (physical, mechanical, chemical and biological) over time. It must retain its functions of use as well as its aesthetic appearance. To ensure this durability, various characterisation methods are used. It was previously stated that the incorporation of biomass ash in concrete affects its properties in the fresh and hardened state, so it is relevant to look at the evolution of its durability properties.

#### 3.4.1. Porosity and Water Absorption

**Figure 12:**
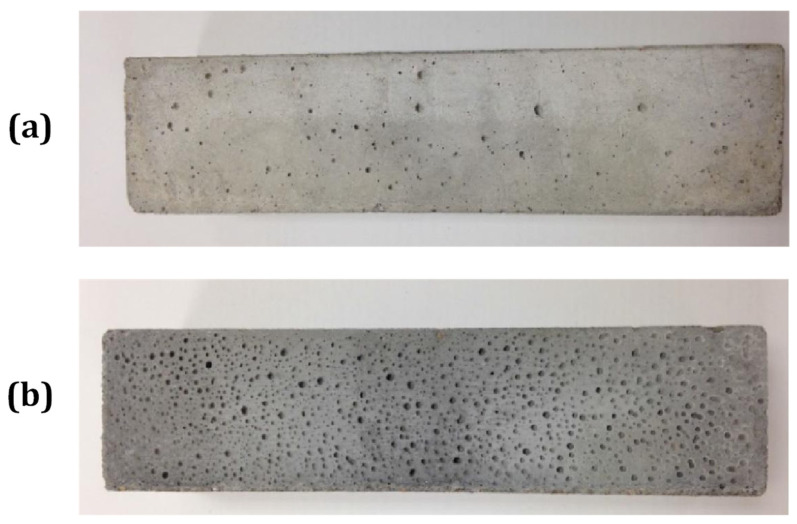
Surface porosity of mortars: (**a**) reference mortar, (**b**) mortar incorporating biomass ash (from [63]).

The penetration of external agents into concrete is largely a function of the pore network of concrete. Several studies on concrete [61], lightweight concrete [55] and self-compacting concrete [40,71] present the results of the water absorption of specimens for substitution rates of 3 to 30% of biomass ash. In general, an increase in water absorption is observed with increasing ash substitution. The water-accessible porosity values are also 5–9% higher compared to the reference mortars formulated with 10–20% BBA instead of sand for Beltrán et al. (2016) [63] and 18–60% higher with 10% BBA for Rosales et al. (2023) [44]. The relative increase in the water absorption of concrete varies from 5 to 128% depending on the study. The difference in the source of the ash and the initial properties of the concrete may explain these changes, although the trend is towards an increase in the water absorption of the concrete. The wide variety of morphologies and the porous nature of the particles contained in the biomass ash play a major role in its water absorption, which will modify the amount of water absorbed and the porosity of the specimens. The origin of the biomass (wood, agricultural, pulp and paper industry) influences the chemical composition of the ash [39] and also the proportion of loss on ignition in the ash [74]. This can negatively affect the porosity when BBA is incorporated. The surface porosity is also affected when using BA [26,63], which can be a limiting factor in the case of facing concrete (Figure 12). In the case of architecturally restricted surfaces, the use of biomass ash may be restricted by these surface effects.

#### 3.4.2. Ultrasonic Pulse Velocity

The ultrasonic pulse velocity can be used as an indicator of the quality and homogeneity of concrete in relation to its density. It is based on the measurement of the propagation time of a high-frequency sound wave through the material, which is carried out according to EN 12504-4 [75]. The effects of BBA substitution on the ultrasonic pulse velocity in concrete have been studied by different authors [54,55,66,70]. The authors show that the incorporation of BBA as sand in concrete decreases the ultrasonic pulse velocity. These decreases could be correlated to the reduction in compressive strength. They are generally greater when the substitution rate of BBA increases. This indicator confirms that the incorporation of BBA tends to reduce the quality of concrete at high rates of upgrading.

#### 3.4.3. Depth of Carbonation

**Figure 13:**
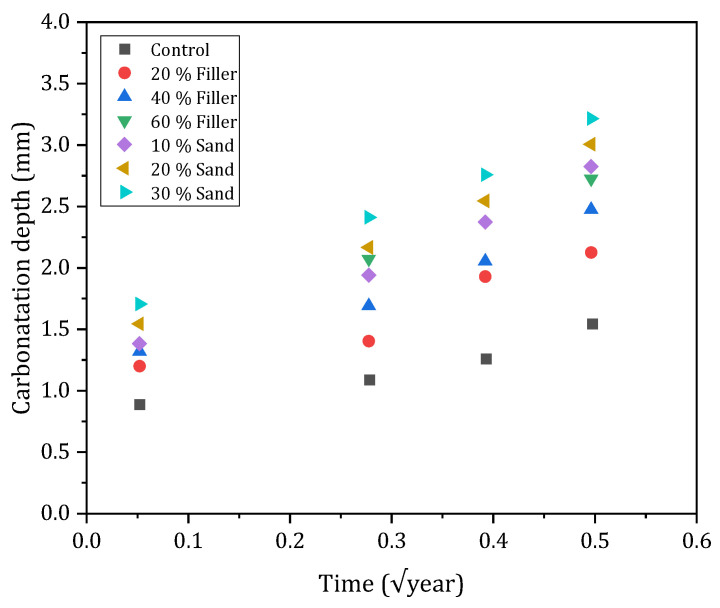
Carbonation depth [40].

The phenomenon of carbonation in cementitious materials is a critical parameter associated with the corrosion of reinforcing steels. It is dependent on the porosity and permeability properties of these materials. Thus, as previously discussed, the increase in porosity associated with the addition of BA into the mix may intensify the effects of carbonation. The study of Cabrera et al. (2021) on self-compacting concretes incorporating 20–30% ash substitution for sand and 20–60% filler substitution shows that the depth of carbonation increases with the ash content (Figure 13). Indeed, the substitution of BA has the effect of doubling the depth of carbonation during accelerated carbonation tests (HR 55–65%; T 23 ± 3 °C; CO_2_ 5 ± 0.1%) over 3 months. Similar results are observed in the work of Cuenca-Moyano et al. (2023) [54]. These results are in line with the increase in porosity and the decrease in the mechanical strength of concretes incorporating BA [40].

#### 3.4.4. Penetration of Chloride Ions

The penetration of chloride ions into the concrete leads to the risk of the depassivation of reinforcing steels. In Table 2, you can find the results of two studies that have been reported in the literature. Beltrán et al. (2014) studied the properties of concretes incorporating 3–6% ash in substitution of sand. As part of their studies, they performed chloride ion penetration tests. An increase of 1.9 to 2.1 times the reference value was observed for the penetration depth at 28 days of curing for 3 and 6% substitution, respectively. After 56 days of curing, the penetration depth was 28 to 50% greater than the reference when ash was incorporated [61].

Agrela et al. (2017) also studied chloride ion penetration on concretes with higher enhancement rates ranging from 15 to 30% and for untreated and treated BBA. After 56 days of curing, formulations incorporating 15–30% BBA showed 1.6–1.9 times higher chloride ion penetration than the reference formulation, with or without BBA treatment [66]. The porosity of the particles, as well as the high presence of organic matter in BBA, decreases the quality of the concrete by increasing the porosity, which reduces the resistance to chloride ion penetration of concretes incorporating BBA.

#### 3.4.5. Shrinkage

**Figure 14:**
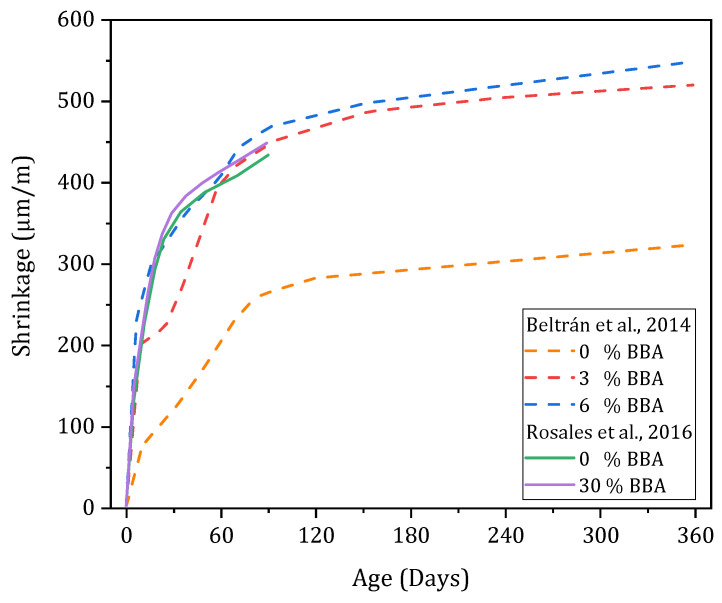
Evolution of shrinkage according to the literature [55,61].

Drying shrinkage at the concrete scale has been studied in several research studies [55,61,66]. The different studies show significant increases in shrinkage with the incorporation of BBA (Figure 14). The relative increase in shrinkage of formulations containing BBA is 71–74% with 3–6% BBA, respectively, for the study of Beltrán et al. (2014) and 45–76% with 15–30% BBA, respectively, for the study of Agrela et al. (2017) after 180 days of curing. The incorporation of 10% BBA to replace sand in mortars in another study led to an increase from 1.5 to 2.5 in the reference value after 90 days [61]. The presence of SO_3_ in biomass ash (0.28–0.49%), although below the limit values of EN 450-1, can potentially influence the shrinkage of the cement paste. The decrease in the W/C ratio when incorporating biomass ash due to its water absorption also tends to increase the drying shrinkage of concrete. The presence of unbrushed elements, which are very friable and porous, can also favour shrinkage. Similar findings have been observed when producing lightweight foam concrete incorporating palm oil ash [76].

## 4. Conclusions

The examination of the properties of biomass ash and the cementitious materials formulated with its incorporation lead to the following conclusions.

- Many studies have characterised the properties of biomass ash (fly ash and bottom ash). The high water absorption and the presence of organic matter are limiting factors for their reuse. Their amorphous nature and granulometry make them interesting for granular recovery. However, few studies have addressed the treatment of ash to improve its properties. It might be useful to specify the combustion technology, which is not always the case, as this would help to explain some of the significant variations found between studies—particularly when the biomass source is similar but the combustion technology is different.
- The density of BBA varies significantly in the literature, from 1.73 to 2.69 g/cm^3^, which is generally lower than that of sand. To avoid introducing errors into the studies, it is important to carry out the volume substitution of the material when replacing sand with BBA.
- Several works incorporate up to 30% ash in different types of concrete. The workability of the concrete is generally maintained through the use of a superplasticiser. The density of the concrete produced decreases with the rate of substitution due to the lower density of BBA compared to a common aggregate. A reduction in mechanical strength is also observed with the incorporation of biomass ash and may be related to the high water absorption of BBA.
- The durability properties of concrete incorporating biomass ash are poorly studied in the literature, except for the open porosity properties, which show a decrease with an increasing BBA substitution rate. Indicators of carbonation resistance and chloride ion penetration have been characterised in the literature, but there are few results available to confirm a trend for these ashes.
- The compatibility of biomass ash with steel for reinforced concrete is not studied in the literature.

To improve our understanding of the properties of BBA, the combustion technology should be cited in the research work. The presence of heavy metals is also not systematically studied and should be examined when recovering BBA—particularly when granular recovery is envisaged, as the quantity of material will be greater than for cement recovery. It may be relevant to develop research on bottom ash from fluidised bed technology. This is because it is largely composed of sand from the combustion technology, which could facilitate its granular recovery. There is also very little literature on the subject, although this technology is developing, which implies an increase in the volume of waste produced and therefore needing to be disposed of or recycled. Biomass ash could also be an interesting filler for building materials. As highlighted in this article, the absence of reactivity of biomass ash makes it an ideal filler. Investigations are still needed to document the databases, particularly with regard to the durability properties of cementitious materials. The parameters required for the numerical modelling of the effects of ash in concrete formulations are also poorly studied, although they could facilitate the valorisation of ash. Further work is needed to incorporate ash into standards and to encourage a move to an industrial scale.

## Figures and Tables

**Table 2:** Penetration properties of chloride ions (mm) [61].

Author	Age (Days)	Rate of Valorisation (%)	Chloride Diffusion (mm)
[61]	28	0	10.3
		3	19.7
		6	21.8
	56	0	18.1
		3	23.1
		6	27.2
[66]	56	0	41.8
		15	74.6
		15 Pr *	70.1
		30	81.6
		30 Pr *	79.9

* Pr: Processed BBA.

## Data Availability

Data will be made available on request.

## References

[1] European Parliament and Council of the European Union Directive 2009/28/EC of the European Parliament and of the Council of 23 April 2009 on the Promotion of the Use of Energy from Re-Newable Sources. https://eur-lex.europa.eu/legal-content/EN/ALL/?uri=celex%3A32009L0028.

[2] European Parliament and Council of the European Union Directive (EU) 2018/2001 of the European Parliament and of the Council of 11 December 2018 on the Promotion of the Use of Energy from Renewable Sources. https://eur-lex.europa.eu/eli/dir/2018/2001/oj.

[3] Zhai J., Burke I.T., Stewart D.I. (2021). Beneficial Management of Biomass Combustion Ashes. Renew. Sustain. Energy Rev..

[4] Carević I., Serdar M., Štirmer N., Ukrainczyk N. (2019). Preliminary Screening of Wood Biomass Ashes for Partial Resources Replacements in Cementitious Materials. J. Clean. Prod..

[5] Pitman R.M. (2006). Wood Ash Use in Forestry—A Review of the Environmental Impacts. For. Int. J. For. Res..

[6] Bendixen M., Best J., Hackney C., Iversen L.L. (2019). Time Is Running out for Sand. Nature.

[7] United Nations Environment Programme (2019). Sand and Sustainability: Finding New Solutions for Environmental Governance of Global Sand Resources.

[8] Sverdrup H.U., Koca D., Schlyter P. (2017). A Simple System Dynamics Model for the Global Production Rate of Sand, Gravel, Crushed Rock and Stone, Market Prices and Long-Term Supply Embedded into the WORLD6 Model. Biophys. Econ. Sustain..

[9] Akhtar M.N., Bani-Hani K.A., Malkawi D.A.H., Albatayneh O. (2024). Suitability of Sustainable Sand for Concrete Manufacturing—A Complete Review of Recycled and Desert Sand Substitution. Results Eng..

[10] Zhong X., Deetman S., Tukker A., Behrens P. (2022). Increasing Material Efficiencies of Buildings to Address the Global Sand Crisis. Nat. Sustain..

[11] United Nations Environment Programme (2014). Sand, Rarer than One Thinks.

[12] Eurostat (European Commission) (2021). Key Figures on Europe: 2022 Edition.

[13] (2012). Cement—Part 1: Composition, Specifications and Conformity Criteria for Common Cements.

[14] (2012). Fly Ash for Concrete—Part 1: Definition, Specifications and Conformity Criteria.

[15] Muthusamy K., Rasid M.H., Jokhio G.A., Mokhtar Albshir Budiea A., Hussin M.W., Mirza J. (2020). Coal Bottom Ash as Sand Replacement in Concrete: A Review. Constr. Build. Mater..

[16] Singh N., Shehnazdeep, Bhardwaj A. (2020). Reviewing the Role of Coal Bottom Ash as an Alternative of Cement. Constr. Build. Mater..

[17] Cabrera M., Díaz-López J.L., Agrela F., Rosales J. (2020). Eco-Efficient Cement-Based Materials Using Biomass Bottom Ash: A Review. Appl. Sci..

[18] Katare V.D., Madurwar M.V. (2017). Experimental Characterization of Sugarcane Biomass Ash—A Review. Constr. Build. Mater..

[19] Koppejan J., Van Loo S. (2007). The Handbook of Biomass Combustion and Co-Firing.

[20] Mandø M. (2013). Direct Combustion of Biomass. Biomass Combustion Science, Technology and Engineering.

[21] Anicic B., Lin W., Dam-Johansen K., Wu H. (2018). Agglomeration Mechanism in Biomass Fluidized Bed Combustion—Reaction between Potassium Carbonate and Silica Sand. Fuel Process. Technol..

[22] Gatternig B., Karl J. (2015). Investigations on the Mechanisms of Ash-Induced Agglomeration in Fluidized-Bed Combustion of Biomass. Energy Fuels.

[23] Morris J., Daood S., Chilton S., Nimmo W. (2018). Mechanisms and Mitigation of Agglomeration during Fluidized Bed Combustion of Biomass: A Review. Fuel.

[24] Visser H. The Influence of Fuel Composition on Agglomeration Behaviour in Fluidised Bed Combustion. https://publications.tno.nl/publication/34628434/5f7Iz3/c04054.pdf.

[25] Schlupp F., Page J., Djelal C., Libessart L. (2023). Influence of Recycled Sand from Biomass Combustion on the Mechanical, Hydration and Porous Properties of Mortar Mixtures. Constr. Build. Mater..

[26] Lessard J.-M., Omran A., Tagnit-Hamou A., Gagne R. (2017). Feasibility of Using Biomass Fly and Bottom Ashes in Dry-Cast Concrete Production. Constr. Build. Mater..

[27] Li L., Yu C., Bai J., Wang Q., Luo Z. (2012). Heavy Metal Characterization of Circulating Fluidized Bed Derived Biomass Ash. J. Hazard. Mater..

[28] Hussin M.W., Muthusamy K., Zakaria F. (2010). Effect of Mixing Constituent toward Engineering Properties of POFA Cement-Based Aerated Concrete. J. Mater. Civ. Eng..

[29] Jamil M., Kaish A.B.M.A., Raman S.N., Zain M.F.M. (2013). Pozzolanic Contribution of Rice Husk Ash in Cementitious System. Constr. Build. Mater..

[30] Awang H.B., Al-Mulali M.Z., Waisundara V. (2018). The Inclusion of Palm Oil Ash Biomass Waste in Concrete: A Literature Review. Palm Oil.

[31] Sales A., Lima S.A. (2010). Use of Brazilian Sugarcane Bagasse Ash in Concrete as Sand Replacement. Waste Manag..

[32] Omran A., Soliman N., Xie A., Davidenko T., Tagnit-Hamou A. (2018). Field Trials with Concrete Incorporating Biomass-Fly Ash. Constr. Build. Mater..

[33] Rajamma R. (2011). Biomass Fly Ash Incorporation in Cement Based Materials. Ph.D. Thesis.

[34] Rajamma R., Senff L., Ribeiro M.J., Labrincha J.A., Ball R.J., Allen G.C., Ferreira V.M. (2015). Biomass Fly Ash Effect on Fresh and Hardened State Properties of Cement Based Materials. Compos. Part B Eng..

[35] Cuenca J., Rodríguez J., Martín-Morales M., Sánchez-Roldán Z., Zamorano M. (2013). Effects of Olive Residue Biomass Fly Ash as Filler in Self-Compacting Concrete. Constr. Build. Mater..

[36] Vassilev S.V., Baxter D., Andersen L.K., Vassileva C.G. (2013). An Overview of the Composition and Application of Biomass Ash. Part 1. Phase–Mineral and Chemical Composition and Classification. Fuel.

[37] Magdziarz A., Dalai A.K., Koziński J.A. (2016). Chemical Composition, Character and Reactivity of Renewable Fuel Ashes. Fuel.

[38] Tarelho L.A.C., Teixeira E.R., Silva D.F.R., Modolo R.C.E., Labrincha J.A., Rocha F. (2015). Characteristics of Distinct Ash Flows in a Biomass Thermal Power Plant with Bubbling Fluidised Bed Combustor. Energy.

[39] Vassilev S.V., Baxter D., Andersen L.K., Vassileva C.G. (2010). An Overview of the Chemical Composition of Biomass. Fuel.

[40] Cabrera M., Martinez-Echevarria M.J., López-Alonso M., Agrela F., Rosales J. (2021). Self-Compacting Recycled Concrete Using Biomass Bottom Ash. Materials.

[41] Grau F., Choo H., Hu J.W., Jung J. (2015). Engineering Behavior and Characteristics of Wood Ash and Sugarcane Bagasse Ash. Materials.

[42] Modolo R.C.E., Ferreira V.M., Tarelho L.A., Labrincha J.A., Senff L., Silva L. (2013). Mortar Formulations with Bottom Ash from Biomass Combustion. Constr. Build. Mater..

[43] Sklivaniti V., Tsakiridis P.E., Katsiotis N.S., Velissariou D., Pistofidis N., Papageorgiou D., Beazi M. (2017). Valorisation of Woody Biomass Bottom Ash in Portland Cement: A Characterization and Hydration Study. J. Environ. Chem. Eng..

[44] Rosales M., Agrela F., Sánchez de Rojas M.I., Cabrera M., Rosales J. (2023). Optimisation of Hybrid Eco-Efficient Mortars with Aggregates from Construction and Demolition Waste and Olive Biomass Ash. Constr. Build. Mater..

[45] Rosales, Cabrera M., Beltrán M.G., López M., Agrela F. (2017). Effects of Treatments on Biomass Bottom Ash Applied to the Manufacture of Cement Mortars. J. Clean. Prod..

[46] Rissanen J., Ohenoja K., Kinnunen P., Romagnoli M., Illikainen M. (2018). Milling of Peat-Wood Fly Ash: Effect on Water Demand of Mortar and Rheology of Cement Paste. Constr. Build. Mater..

[47] Schlupp F., Page J., Djelal C., Libessart L. (2023). Use of Biomass Bottom Ash as Granular Substitute in Mortar. J. Build. Eng..

[48] Lothenbach B., Scrivener K., Hooton R.D. (2011). Supplementary Cementitious Materials. Cem. Concr. Res..

[49] Thy P., Jenkins B.M., Grundvig S., Shiraki R., Lesher C.E. (2006). High Temperature Elemental Losses and Mineralogical Changes in Common Biomass Ashes. Fuel.

[50] Ukrainczyk N., Vrbos N., Koenders E.A.B. (2016). Reuse of Woody Biomass Ash Waste in Cementitious Materials. Chem. Biochem. Eng. Q..

[51] Teixeira E.R., Camões A., Branco F.G. (2019). Valorisation of Wood Fly Ash on Concrete. Resour. Conserv. Recycl..

[52] Hinojosa M.J.R., Galvín A.P., Agrela F., Perianes M., Barbudo A. (2014). Potential Use of Biomass Bottom Ash as Alternative Construction Material: Conflictive Chemical Parameters According to Technical Regulations. Fuel.

[53] Sow M., Hot J., Tribout C., Cyr M. (2015). Characterization of Spreader Stoker Coal Fly Ashes (SSCFA) for Their Use in Cement-Based Applications. Fuel.

[54] Cuenca-Moyano G.M., Cabrera M., López-Alonso M., Martínez-Echevarría M.J., Agrela F., Rosales J. (2023). Design of Lightweight Concrete with Olive Biomass Bottom Ash for Use in Buildings. J. Build. Eng..

[55] Rosales J., Beltrán M.G., Cabrera M., Velasco A., Agrela F. (2016). Feasible Use of Biomass Bottom Ash as Addition in the Manufacture of Lightweight Recycled Concrete. Waste Biomass Valorization.

[56] Carević I., Štirmer N., Trkmić M., Kostanić Jurić K. (2020). Leaching Characteristics of Wood Biomass Fly Ash Cement Composites. Appl. Sci..

[57] Tosti L., van Zomeren A., Pels J.R., Dijkstra J.J., Comans R.N.J. (2019). Assessment of Biomass Ash Applications in Soil and Cement Mortars. Chemosphere.

[58] Jura J., Ulewicz M. (2021). Assessment of the Possibility of Using Fly Ash from Biomass Combustion for Concrete. Materials.

[59] Kim H.K., Jeon J.H., Lee H.K. (2012). Flow, Water Absorption, and Mechanical Characteristics of Normal- and High-Strength Mortar Incorporating Fine Bottom Ash Aggregates. Constr. Build. Mater..

[60] Rafieizonooz M., Mirza J., Salim M.R., Hussin M.W., Khankhaje E. (2016). Investigation of Coal Bottom Ash and Fly Ash in Concrete as Replacement for Sand and Cement. Constr. Build. Mater..

[61] Beltrán M.G., Agrela F., Barbudo A., Ayuso J., Ramírez A. (2014). Mechanical and Durability Properties of Concretes Manufactured with Biomass Bottom Ash and Recycled Coarse Aggregates. Constr. Build. Mater..

[62] Cabrera M., Agrela F., Ayuso J., Galvin A.P., Rosales J. (2016). Feasible Use of Biomass Bottom Ash in the Manufacture of Cement Treated Recycled Materials. Mater. Struct..

[63] Beltrán M.G., Barbudo A., Agrela F., Jiménez J.R., de Brito J. (2016). Mechanical Performance of Bedding Mortars Made with Olive Biomass Bottom Ash. Constr. Build. Mater..

[64] Cabrera M., Galvin A.P., Agrela F., Carvajal M.D., Ayuso J. (2014). Characterisation and Technical Feasibility of Using Biomass Bottom Ash for Civil Infrastructures. Constr. Build. Mater..

[65] (2008). Aggregates for Concrete.

[66] Agrela F., Beltran M.G., Cabrera M., López M., Rosales J., Ayuso J. (2017). Properties of Recycled Concrete Manufacturing with All-in Recycled Aggregates and Processed Biomass Bottom Ash. Waste Biomass Valorization.

[67] da Costa T.P., Quinteiro P., Tarelho L.A.C., Arroja L., Dias A.C. (2019). Environmental Assessment of Valorisation Alternatives for Woody Biomass Ash in Construction Materials. Resour. Conserv. Recycl..

[68] Berra M., Mangialardi T., Paolini A.E. (2015). Reuse of Woody Biomass Fly Ash in Cement-Based Materials. Constr. Build. Mater..

[69] Modolo R.C.E., Silva T., Senff L., Tarelho L.A.C., Labrincha J.A., Ferreira V.M., Silva L. (2015). Bottom Ash from Biomass Combustion in BFB and Its Use in Adhesive-Mortars. Fuel Process. Technol..

[70] Sua-iam G., Makul N. (2013). Use of Increasing Amounts of Bagasse Ash Waste to Produce Self-Compacting Concrete by Adding Limestone Powder Waste. J. Clean. Prod..

[71] Hamza Hasnain M., Javed U., Ali A., Saeed Zafar M. (2021). Eco-Friendly Utilization of Rice Husk Ash and Bagasse Ash Blend as Partial Sand Replacement in Self-Compacting Concrete. Constr. Build. Mater..

[72] Barbosa R., Lapa N., Dias D., Mendes B. (2013). Concretes Containing Biomass Ashes: Mechanical, Chemical, and Ecotoxic Performances. Constr. Build. Mater..

[73] (2021). Testing Hardened Concrete—Part 13: Determination of Secant Modulus of Elasticity in Compression.

[74] Obernberger I. (1998). Nutzung Fester Biomasse in Verbrennungsanlagen: Unter Besonderer Berücksichtigung Des Verhaltens Aschebildender Elemente. Schriftenreihe Thermische Biomassenutzung.

[75] (2021). Testing Concrete in Structures—Part 4: Determination of Ultrasonic Pulse Velocity.

[76] Al-Shwaiter A., Awang H., Khalaf M.A. (2022). Performance of Sustainable Lightweight Foam Concrete Prepared Using Palm Oil Fuel Ash as a Sand Replacement. Constr. Build. Mater..

